# Effective *Agrobacterium*-mediated transformation protocols for callus and roots of halophyte ice plant (*Mesembryanthemum crystallinum*)

**DOI:** 10.1186/s40529-018-0249-3

**Published:** 2019-01-07

**Authors:** Hau-Hsuan Hwang, Chih-Hao Wang, Hsiao-Huei Chen, Jia-Fang Ho, Shin-Fei Chi, Fan-Chen Huang, Hungchen Emilie Yen

**Affiliations:** 1 Department of Life Sciences, National Chung Hsing University, No. 145, Xingda Road, Taichung, 402 Taiwan; 2 Ph.D. Program in Microbial Genomics, National Chung Hsing University and Academia Sinica, Taichung, Taiwan; 3 Ph.D. Program in Microbial Genomics, National Chung Hsing University, Taichung, Taiwan; 4 Advanced Plant Biotechnology Center, National Chung Hsing University, Taichung, Taiwan; 5 Innovation and Development Center of Sustainable Agriculture, National Chung Hsing University, Taichung, Taiwan

**Keywords:** *Agrobacterium*, Transformation protocol, Ice plant, *Mesembryanthemum crystallinum*

## Abstract

**Background:**

Ice plant (*Mesembryanthemum crystallinum* L.) is a model plant for studying salt-tolerant mechanisms in higher plants. Many salt stress-responsive ice plant genes have been identified with molecular and biochemical approaches. However, no further functional characterization of these genes in host plant due to lack of easy and effective transformation protocols.

**Results:**

To establish efficient transformation system of ice plants, three types of ice plant materials, hypocotyl-derived callus, aseptically-grown seedlings and pot-grown juvenile plants, were used to develop *Agrobacterium*-mediated transformation protocols. The highest transient transformation efficiency was with 5-day-old ice plant callus co-incubated with an *Agrobacterium tumefaciens* at 2.5 × 10^9^ cells mL^−1^ for 48 h. The 3-day-old ice plant seedlings with root tip removed were successfully infected with *A. tumefaciens* or *A. rhizogenes*, and obtained 85% and 33–100% transient transformation rates, respectively. The transient transformation assays in ice plant callus and seedlings demonstrated that the concentrations of *Agrobacteria*, the durations of co-incubation time, and the plant growth stages were three important factors affecting the transient transformation efficiencies. Additionally, pot-grown juvenile plants were syringe-injected with two *A. rhizogenes* strains A8196 and NCPPB 1855, to establish transformed roots. After infections, ice plants were grown hydroponically and showed GUS expressions in transformed roots for 8 consecutive weeks.

**Conclusions:**

Our *Agrobacterium*-mediated transformation protocols utilized hypocotyl-derived callus and seedlings as plant materials, which can be easily obtained in large quantity. The average successful transient transformation rates were about 2.4–3.0% with callus and 33.3–100.0% with seedlings. We also developed a rapid and efficient protocol to generate transgenic roots by *A. rhizogenes* infections without laborious and challenging tissue culture techniques. This protocol to establish composite ice plant system demonstrates excellent improvements in efficiency, efficacy, and ease of use over previous ice plant transformation protocols. These *Agrobacterium*-mediated transformation protocols can be versatile and efficient tools for exploring gene functions at cellular and organ levels of ice plants.

**Electronic supplementary material:**

The online version of this article (10.1186/s40529-018-0249-3) contains supplementary material, which is available to authorized users.

## Background

The *Mesembryanthemum crystallinum* L. (family: Aizoaceae, order: Caryophyllales), also known as the common ice plant or the crystalline ice plant, is an important model plant to study the plant response to various environmental abiotic stresses. The average life cycle of *M. crystallinum* is 4–5 months and can be characterized as five distinct growth phases: seedling, juvenile, adult, flowering, and seed-forming stages (Adams et al. 1998). The growth period can be significantly affected by several environmental factors, including water supply, temperature, light quantity and quality, and nutrient supply (Bohnert and Cushman 2000). The genome size of *M. crystallinum* is about 390 Mb distributed in nine chromosomes (DeRocher et al. 1990; Meyer et al. 1990). Because of the relatively small size genome, self-fertilization, and large seed production, *M. crystallinum* is a potential genetic model.

*Mesembryanthemum crystallinum* is a facultative halophyte with distinctive ability to change from C3 photosynthesis to Crassulacean acid metabolism (CAM) under stress, tolerate high salinity by transporting sodium into vacuoles of specialized epidermal bladder cells (EBCs) and accumulate osmolytes in the cytosol during water deficit and salt stress (Bohnert and Cushman 2000; Cushman and Borland 2002). CAM induction in *M. crystallinum* provides a prototype for plant scientists to study various gene and enzyme functions associated with the CAM pathway, such as CAM-specific isoform of phosphoenolpyruvate carboxylase (PEPC) (Cushman et al. 1989; Winter and Holtum 2014).

EBCs are non-glandular (non-secreting) trichomes located on the surface of leaves, stems, and flower buds (Adams et al. 1998). EBCs are underdeveloped and flattened to surfaces of the young plant aerial parts but are enlarged and filled with liquid in adult plants when plants are switching to the CAM photosynthetic pathway and exposed to salt stress. EBCs primarily function in water, sodium, and chloride ion storage when plants have limited water supply or are under salt stress (Rygol et al. 1989; Adams et al. 1998; Jou et al. 2007; Barkla et al. 2016). Recent quantitative proteomic studies demonstrated that several transport proteins and proteins involved in photosynthesis, primary metabolism, and CAM are significantly increased in EBCs, which further supports the important roles of EBCs for salt tolerance in *M. crystallinum* (Barkla et al. 2016). Several transcriptomic, proteomic, and mutant (CAM-less and EBC-less) studies have identified several *M. crystallinum* proteins that play important roles in the CAM pathway and salinity stress responses (Kore-eda et al. 2004; Jou et al. 2007; Barkla et al. 2009, 2012, 2016; Haider et al. 2012; Cosentino et al. 2013; Oh et al. 2015; Tsukagoshi et al. 2015; Chiang et al. 2016).

Besides the well-characterized genes expressed in EBCs and CAM, the functions of many salt stress-responsive genes remain to be characterized due to the lack of available mutants or efficient transformation systems (Bohnert and Cushman 2000). *A. tumefaciens* is a plant pathogen that can transfer its own genetic material into plant cells. The *Agrobacterium*-mediated transfer of genes into plant cells is the most frequently used genetic transformation method and has a number of advantages. It is easy to use and relatively inexpensive and generally results in a low copy number of relatively large fragments of T-DNA insertions as compared with other plant transformation methods, such as particle bombardment (Anami et al. 2013; Hwang et al. 2017). *Agrobacterium tumefaciens*-mediated transformation systems have been attempted by using young leaf, root, cotyledon, hypocotyl, and callus of ice plant, but successful transformation callus was only obtained with root and hypocotyl explants (Ishimaru 1999). Similarly, *Agrobacterium rhizogenes* was used to transform ice plant seedlings and obtained transgenic roots with 6–20% transformation rates (Andolfatto et al. 1994; Konieczny et al. 2011). In addition, very limited established protocols and relatively low efficiencies for whole-plant regeneration from ice plant callus by somatic embryogenesis and organogenesis were reported (Meiners et al. 1991; Cushman et al. 2000; Libik et al. 2005). With the help of plant hormones, the regeneration efficiency increased by using cotyledonary node explants; however, the transformation efficiency with *A. tumefaciens* was only 0.3% (Sunagawa et al. 2007). The hairy-root and transgenic callus cultures from ice plants have been obtained by *A. rhizogenes* and *A. tumefaciens*-mediated transformation for more than 20 years, no any functional study of CAM induction or salt tolerance has been applied, may be due to the low transformation efficiencies. Therefore, there is still a room for improvement of transformation methods for ice plants to facilitate functional genomic studies (Andolfatto et al. 1994; Ishimaru 1999; Sunagawa et al. 2007; Konieczny et al. 2011).

Because of relatively few successful reports regarding ice plant transformation protocols, here we used in vitro-cultured callus and intact plants to establish transformation procedures of ice plants. We established transformation systems of three types of ice plant materials with infection by two kinds of *Agrobacterium* strains, *A. tumefaciens* and *A. rhizogenes*, with high transformation rates which will provide the plant research community with more effective tools to study gene functions in ice plants.

## Methods

### Bacteria strains, plasmids, and culture conditions

*Agrobacterium tumefaciens* and *A. rhizogenes* strains were grown in 523 media (Kado and Heskett 1970) or on 523 agar supplemented with appropriate antibiotics (rifampicin 50 μg mL^−1^, spectinomycin 100 μg mL^−1^, and kanamycin 20 μg mL^−1^) at 28 °C. *Escherichia coli* strains were grown at 37 °C in Luria Broth (LB) or 2× YT media (1.6% tryptone, 1% yeast extract, 0.5% NaCl, pH 7.0) containing appropriate antibiotics (spectinomycin 100 μg mL^−1^ and kanamycin 20 μg mL^−1^). A complete list of bacteria strains and plasmids used in this study is in Additional file 1: Table S1.

### Plant materials

Seeds of ice plant (*M. crystallinum*) (collection of the Known-You Seed Co., Kaohsiung, Taiwan) were surface-sterilized and placed on Murashige and Skoog basal medium (MS medium) (Murashige and Skoog 1962) containing 3% (w/v) sucrose and 1% (w/v) agar. The 3-, 5-, and 7-day-old seedlings grown in constant 50 μmol m^−2^ s^−1^ light at 25 °C were used for transient transformation assays. Additionally, hypocotyl explants of 7-day-old ice plant seedlings were used as initial materials for callus inductions. Callus tissue was initiated in callus-inducing medium (CIM) (20.6 mM NH_4_NO_3_, 18.8 mM KNO_3_, 3 mM CaCl_2_, 1.5 mM MgSO_4_, 2.5 mM KH_2_PO_4_, 100 μM H_3_BO_3_, 100 μM MnSO_4_, 5 μM KI, 30 μM ZnSO_4_, 1 μM NaMoO_4_, 0.1 μM CuSO_4_, 0.1 μM CoCl_2_, 100 μM Fe-EDTA, 560 μM myo-inositol, 8 μM nicotinic acid, 5 μM pyridoxol-HCl, 30 μM thiamine chloride, 0.5 μM kinetin, 23 μM 2,4-dichlorophenoxyacetic acid, 1.5% sucrose, 1% agar, pH 5.6–5.8) under constant 50 μmol m^−2^ s^−1^ light at 25 °C according to Jou et al. (2006). The callus cultures were maintained in same conditions and subcultured every 10–14 days.

Seeds were germinated directly in mixed soil (peat moss: vermiculite: sand 3:1:2) under a 14-h light/10-h dark at 300 μmol m^−2^ s^−1^ and 25 °C. 5 to 6 weeks after germination, juvenile plants were infected with *A. rhizogenes*. One week after infection, plants were grown hydroponically in a modified Johnson’s nutrient solution as described (Chu et al. 1990).

### Transformation assays of hypocotyl-derived callus with *A. tumefaciens*

Assays of transformation of ice plant callus with infection by *A. tumefaciens* strains were as described (Ishimaru 1999) with minor modifications. Bacteria strains were first grown in 523 media with appropriate antibiotics at 28 °C to OD_600_ 0.8–1.0. The bacteria cells were then washed and resuspended in AB-MES media (2% glucose, 0.3% K_2_HPO_4_, 0.03% MgSO_4_·7H_2_O, 0.1% NaH_2_PO_4_, 0.1% NH_4_Cl, 0.015% KCl, 0.001% CaCl_2_, 0.00025% FeSO_4_·7H_2_O, and 0.97% MES, pH 5.5) (Hwang et al. 2010) without antibiotics at 28 °C to mid-log phase for 6 h. After adding 200 μM acetosyringone (AS; Sigma-Aldrich, St. Louis, MO, USA), bacteria cultures were further cultivated at 28 °C for 16–18 h and collected, resuspended in liquid CIM at the desired bacteria concentrations for infection.

The hypocotyl-derived callus was transferred from solid to liquid medium for subsequent *Agrobacterium* infection and GUS staining. During repeatedly shaking and washing of callus, a large quantity of “single cells” was released into the liquid medium and named as “callus-derived cells”. The callus cultures were maintained in 25 mL of liquid CIM medium in 125 mL Erlenmeyer flasks and subcultured every 14 days.

The *A. tumefaciens* EHA105 strain containing the T-DNA binary pBISN1 plasmid (Additional file 1: Table S1) was used to co-incubate with the 5-, 7-, 10-, or 14-day-old callus for 2 days. Two days after co-incubation with bacteria strains, ice plant callus was washed and sonicated for 10 min in liquid CIM medium containing timentin (100 μg mL^−1^) five times to remove bacteria, then transferred into the CIM medium containing timentin (100 μg mL^−1^) and kanamycin (100 μg mL^−1^). More than 2 × 10^5^ ice plant callus-derived cells were infected with *A. tumefaciens* strain for each independent transformation assay. After 1- or 2-day recovery, ice plant callus was stained with X-gluc staining solution [50 mM sodium phosphate buffer, 0.1% Tween 20, 3% sucrose, and 1–2 mM 5-bromo-4-chloro-3-indolyl-β-d-glucuronic acid (X-Gluc), pH 7.0] for 1 day at 37 °C. An Olympus IX71 fluorescence microscope (Olympus Optical Co. Ltd., Tokyo, Japan) with the DP Controller program was used to observe GUS expression in ice plant callus-derived cells and determine transient transformation efficiencies. We treated GUS-stained samples with 0.5% pectinase (macerase) for 1 day to disrupt the aggregates before counting cell number by hemacytometer. More than 5 × 10^3^ callus-derived cells were examined and counted for each transformation experiment to determine transient transformation rates. Transformation rates were mean ± SD (standard deviation) from at least 3 independent experiments. The transformed callus was then continuously cultured in CIM medium containing 100 μg mL^−1^ kanamycin.

### Transformation assays of aseptically-grown seedlings with *A. tumefaciens* and *A. rhizogenes*

Assays of transformation of ice plant seedlings with infection by *A. tumefaciens* and *A. rhizogenes* strains were as described (Andolfatto et al. 1994; Ishimaru 1999; Sunagawa et al. 2007; Konieczny et al. 2011) with minor modifications. Bacteria cultures were prepared as mentioned in the above paragraph and resuspended in MS medium at the desired concentrations for infection. The 3-, 5-, or 7-day-old intact ice plant seedlings or seedlings with root tip removed were co-incubated with 2.5 × 10^9^ cfu (colony forming unit) mL^−1^ of the *A. tumefaciens* EHA105 strain containing the pBISN1 plasmid (Additional file 1: Table S1) or the *A. rhizogenes* A4 and A8196 strains containing a binary vector pCAMBIA1303 (Additional file 1: Table S1) for 2 days. After 2-day co-incubation period, ice plant seedlings were washed with liquid MS medium containing timentin to remove bacteria and transferred to MS medium with timentin for 1, 2, 5, or 7 days. Ice plant seedlings were then stained for GUS activities as described above and were observed with a stereoscopic microscope to determine transient transformation efficiencies. About 60–80 ice plant seedlings were infected with *A. rhizogenes* strain for each independent transformation assay. Data are mean ± SD (standard deviation) from at least 3 independent experiments.

### Transformation assays of pot-grown juvenile plants with *A. rhizogenes*

The 5- to 6-week-old pot-grown ice plants were infected with *A. rhizogenes* strains (Additional file 1: Table S1) according to Hwang et al. (2010) and (2013). Bacteria strains were grown in 523 media with appropriate antibiotics at 28 °C to OD_600_ 0.8–1.0. Bacteria cells were then washed and resuspended in 154 mM NaCl at the 10^9^ cfu mL^−1^ bacteria concentrations for infection. A syringe was used to inject 100 μL bacteria cultures into 5- to 6-week-old plant stems. One week after infection, pot-grown ice plants were transferred to hydroponic culture as described (Chu et al. 1990). Because the optimal growth of halophytic ice plants is achieved with 100–200 mM NaCl in the hydroponic solution, NaCl was added stepwise to reach a final concentration of 200 mM in a 4-week period. Roots of hydroponically grown plants were stained with X-gluc staining solution to examine positive GUS staining.

About 16–20 ice plants were infected with each *A. rhizogenes* strain for each independent transformation assay. The transformation efficiencies were average values from at least three independent experiments. Error bars were calculated using Excel STDEVP function. The significance test between treatments was based on pairwise Student *t*-test, P < 0.05.

### DNA isolation, genomic DNA PCR, and Southern blot analysis

The DNA isolation protocol from ice plant tissues was based on Weigel and Glazebrook (2002) with minor modifications. Liquid nitrogen frozen plant tissues were ground and mixed with extraction buffer (250 mM NaCl, 25 mM EDTA, 0.5% SDS, 200 mM Tris–HCl, pH 7.5). The isolated plant extract was mixed with an equal amount of phenol: chloroform: isoamyl alcohol (25:24:1) solutions to remove proteins, then the supernatant was precipitated with cold isopropanol to isolate genomic DNA. The 100-ng genomic DNA was used for PCR reactions with GeneTaq DNA polymerase (GenePure, Taiwan) and pairs of primers (Additional file 2: Table S2) for the presence of β-glucuronidase (*GUS*), yellow fluorescent protein (*YFP*), kanamycin resistance gene (*Kan*), or phosphoenolpyruvate carboxylase (*PPC1*) in transformed and untransformed plant tissues.

For Southern blot analysis, genomic DNA of root tissues was treated with 10 μg L^−1^ RNase for 4 h at 25 °C followed by phenol–chloroform extraction and ethanol precipitation. Thirty μg DNA of each root sample was cut with *Sph*I-HF (New England Biolabs, Ipswich, MA, USA) for 16 h at 25 °C. The digested products were separated by 1% agarose gel, denatured in 1.5 M NaCl, 0.5 M NaOH for 30 min, and transferred to hybridization membrane (GeneScreen, DuPont, Boston, MA, USA). Hybridization was performed at 65 °C with ^32^P-labeled probe (Amersham Rediprim II, GE Healthcare, Pittsburgh, PA, USA). The probe is a PCR amplified and gel purified product of pCAMBIA1303 using GUS1 and GUS2 primer (Additional file 2: Table S2). After 20 h of hybridization, the membrane was washed twice in 2× SSC at room temperature for 15 min each, twice in 2× SSC, 1% SDS at 65 °C for 30 min each, and finally once in 0.1× SSC at room temperature for 30 min. The membrane was exposed to an imaging plate (BAS-MS, Fuji Film, Tokyo, Japan) at room temperature and signal was detected by a phosphorimager (Typhoon FLA 7000, GE Healthcare, Pittsburgh, PA, USA).

### Protein extraction and Western blot analyses

Ice plant tissues were ground with liquid nitrogen and mixed with CelLytic P (Sigma Chemical Co., St. Louis, MO, USA) supplemented with the 2 mM leupeptin and a protease inhibitor cocktail (1:100 dilution) for plant cell extracts from Sigma (product number: P 9599). Crude plant protein extracts were isolated as the manufacturer instructed. The protein concentrations were determined by using a BCA protein assay kit (Pierce, Rockford, IL) and spectroscopy (SPECTRA MAX PLUS 384 Molecular Devices, Sunnyvale, CA, USA). Equal amounts of plant proteins were loaded on 12.5% SDS-polyacrylamide gels and western blot analysis was performed (Ausubel et al. 2003) with a 1:1000 dilution of anti-GUS antibody (Sigma Chemical Co., St. Louis, MO, USA). Membranes were developed by using a chemiluminescent detection method (Super Signal West Pico Kit from Pierce, Rockford, IL, USA). Proteins were also stained with the Coomassie brilliant blue R-250 to demonstrate equally loaded protein amounts in each lane.

## Results

### Optimization of protocol for *A. tumefaciens*-mediated transient transformation in ice plant callus

**Fig. 1:**
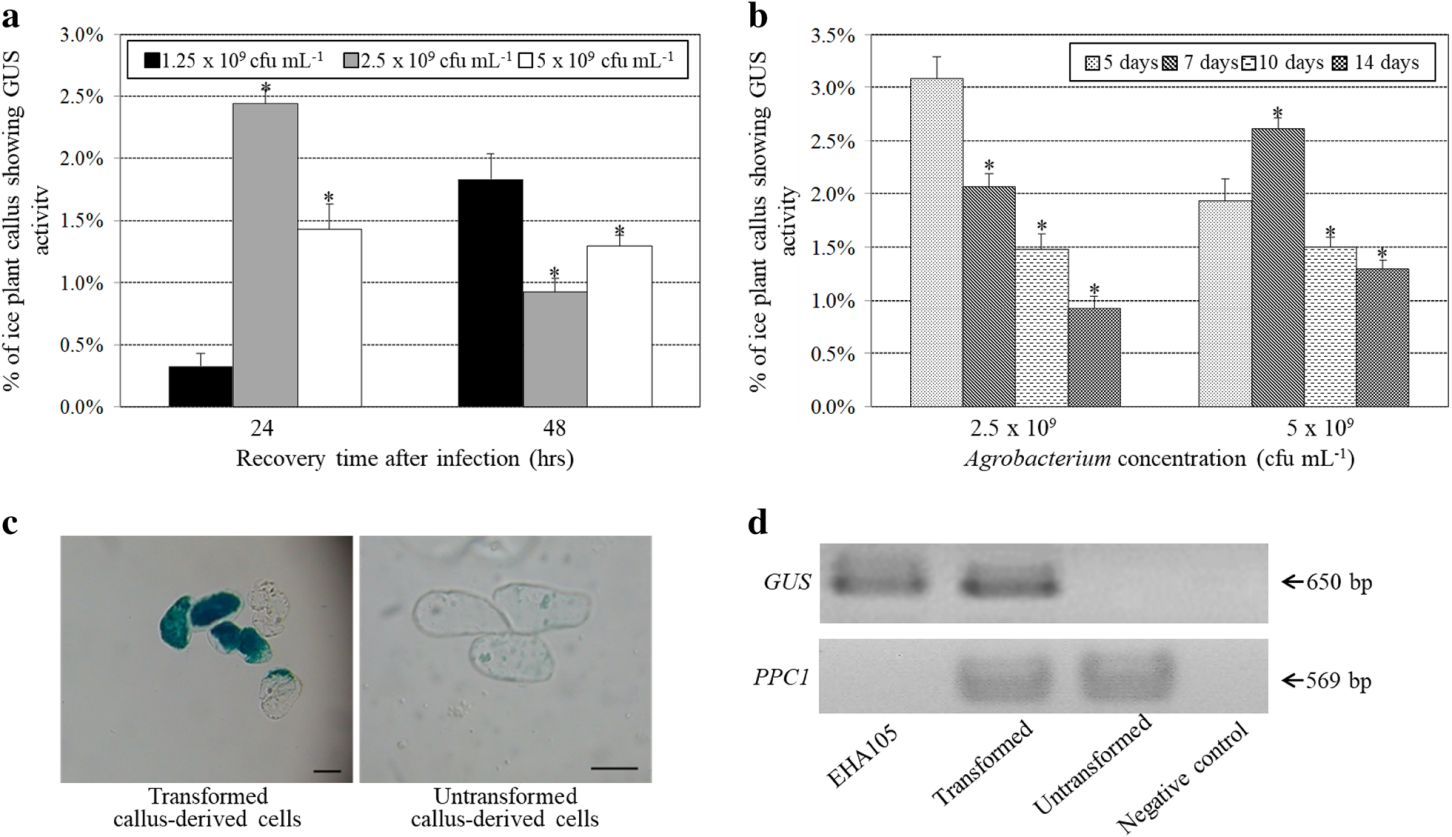
The transient transformation efficiencies of ice plant callus after infection with *Agrobacterium tumefaciens* EHA105 strain harboring the pBISN1 binary vector. **a** Transformation rates of 14-day-old callus co-incubated with 3 bacteria concentration solutions for 48 h and recovered for 24 or 48 h after infections. More than 2 × 10^5^ callus-derived cells were infected with *A. tumefaciens* strain for each independent transformation assay. Data are mean ± SD from at least 3 independent experiments. *P < 0.05 comparing ice plant callus-infected with 2.5 or 5 × 10^9^ cfu mL^−1^ bacteria and callus-infected with 1.25 × 10^9^ cfu mL^−1^ bacteria after same period of recovery time based on pairwise Student *t*-test. **b** Transformation efficiency of callus collected on different days. Callus was co-cultured with *A. tumefaciens* solutions for 48 h and recovered for 24 h after infection. *P < 0.05 comparing 7-, 10-, or 14-day-old infected callus and 5-day-old infected callus under the same bacteria concentration based on pairwise Student *t*-test. **c** Representative results of transformed ice plant callus-derived cells (left panel) showing GUS staining and untransformed callus-derived cells (right panel). Bar = 50 μm. **d** Genomic DNA PCR results of the *GUS* reporter gene and the endogenous ice plant phosphoenolpyruvate carboxylase (*PPC1*) gene in 7-day-old transformed and untransformed callus. The arrow on the right indicates predicted band size. The *A. tumefaciens* EHA105 strain harboring pBISN1 was a positive control. Distilled water was a negative control

Ishimaru (1999) found 54% *A. tumefaciens*-mediated transformation frequencies in root and hypocotyl of ice plant but did not observe any transformed callus. To establish a workable transformation system for ice plant callus, we tested conditions that affect transformation efficiency, including bacteria concentration, recovery time, and age of callus. *A. tumefaciens* EHA105 strain containing a *GUS* reporter gene in the T-DNA binary pBISN1 plasmid was used to infect ice plant callus. Three bacteria concentrations, 1.25 × 10^9^, 2.5 × 10^9^, and 5 × 10^9^ cfu mL^−1^, were chosen to infect 14-day-old ice plant callus for 2 days, then the callus was placed in CIM medium for 24 or 48 h to recover and determine transient transformation rates. Callus infected with 2.5 × 10^9^ cfu mL^−1^ bacteria with 24-h recovery had the transient transformation rate of 2.4% (Fig. 1a). When the higher bacteria concentration, 5 × 10^9^ cfu mL^−1^, was used to infect ice plant callus, the transient transformation efficiency was only 1.4% with 24-h recovery (Fig. 1a). When callus recovered for 48 h under 1.25 × 10^9^ cfu mL^−1^ bacteria concentration, transient transformation efficiency of 1.8% was obtained (Fig. 1a). With higher bacteria concentrations, 2.5 and 5 × 10^9^ cfu mL^−1^, the transient transformation efficiencies were decreased to 0.9–1.3% with 48-h recovery (Fig. 1a). These data suggest that longer recovery time (48-h) for ice plant callus may be beneficial with lower bacteria concentration (1.25 × 10^9^ cfu mL^−1^) used to infect callus. In summary, ice plant callus showed the highest transient transformation efficiency with bacteria infection concentration 2.5 × 10^9^ cfu mL^−1^ for 24-h recovery.

Because different ages of callus may show different competency for *A. tumefaciens* infection, we used 5-, 7-, 10- or 14-day-old callus to be co-incubated with 2.5 or 5 × 10^9^ cfu mL^−1^ bacteria solutions and examined their transformation rates. The highest transformation rate was with 5-day-old callus co-cultured with 2.5 × 10^9^ cfu mL^−1^ and the transformation rates decreased with increasing age of callus (Fig. 1b). These results suggest that younger ice plant callus may be more susceptible to *A. tumefaciens* infection. To increase the percentage of transformed callus, callus was transferred into liquid CIM containing timentin and kanamycin, and continued to grow for 4 passages. The percentage of transformed callus increased from 3 to 70% under continuous antibiotic selective pressure. GUS staining of transformed and untransformed cultured callus-derived cells (Fig. 1c) and genomic DNA PCR (Fig. 1d) both confirmed that *GUS* was expressed in the transformed callus-derived cells but not present in untransformed callus-derived cells.

To further examine the established transformation protocol, we used another binary vector, pBA002, containing the *YFP* reporter gene and the *bar* selectable marker in the *A. tumefaciens* strain. Transformed callus continued to grow in medium supplemented with 25 mg L^−1^ glufosinate ammonium and expressed YFP protein up to 4 months (Additional file 3: Figure S1). Low intrinsic fluorescence was occasionally detected in the cell wall of untransformed callus-derived cells, which interfered with YFP signals. Therefore, we chose detection by GUS staining in subsequent experiments.

### Transient transformation efficiencies of ice plant seedlings with *A. tumefaciens* or *A. rhizogenes* infection

**Fig. 2:**
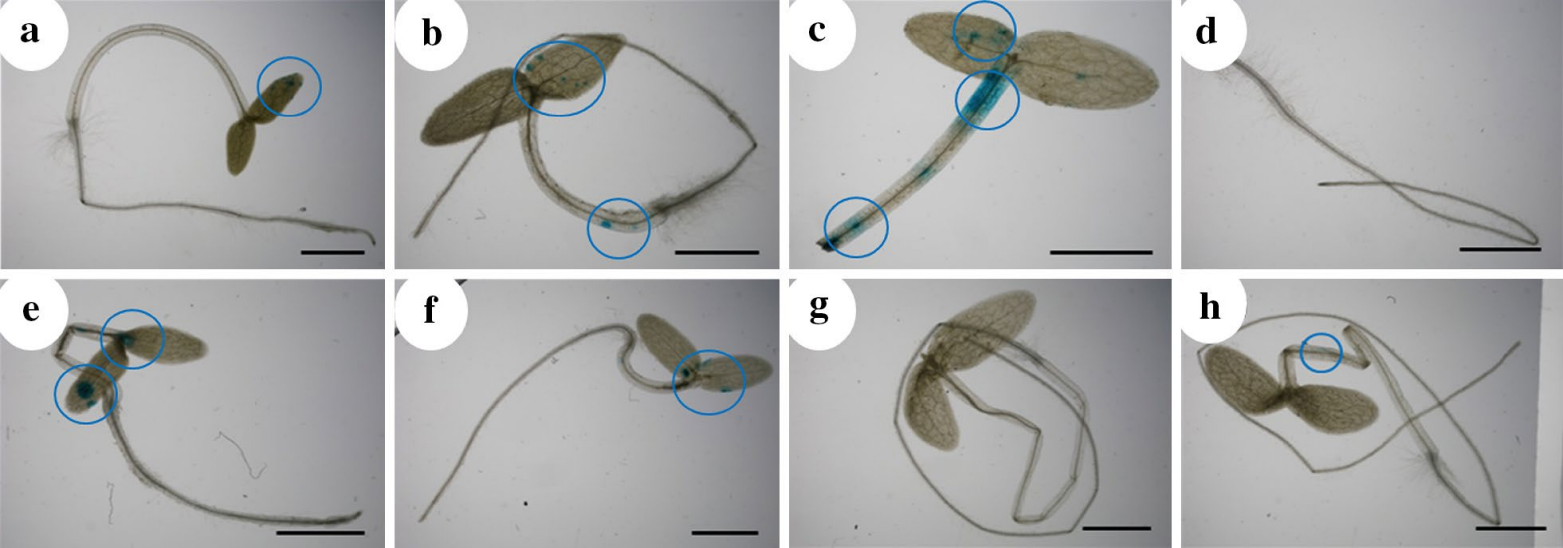
GUS staining results of ice plant seedlings infected with the *A. tumefaciens* EHA105 strain harboring the pBISN1 binary vector. Representative GUS staining results of 5- (**a**–**d**), 3- (**e**, **f**), and 7-day-old (**g**, **h**) seedlings. Blue circles indicate blue spots in intact seedlings (**a**, **e**, **g**); seedlings with root-tip-removed (**b**, **f**, **h**); shoot (**c**) and root (**d**). Tissues showing blue spots indicate successful transformation. Bar = 2 mm

After showing callus can be transformed by *A. tumefaciens*, we used this protocol to test the ability of *A. tumefaciens* infection in intact tissues of ice plant. After 2-day infection and 1-day recovery, 3-, 5-, and 7-day-old seedlings were stained for GUS activities to determine transient transformation efficiency. Representative GUS staining results are in Fig. 2. Among all tested plants, transient transformation efficiency was highest, 85.0%, for 3-day-old plant seedlings with wounding (root tip removed) (Table 1). Similarly, transient transformation rate was higher for intact 3- than 5-day-old seedlings (Table 1). The 7-day-old seedlings had the lowest transient transformation rates, 0.0% and 13.3%, as compared with 3- and 5-day-old seedlings (Table 1). The results suggested that younger tissues with wounding might be more easily infected by *A. tumefaciens*. Transformation results from 3- and 5-day-old seedlings all showed a higher percentage of cotyledons than hypocotyls with GUS activities. Hence, cotyledons might be more susceptible to bacteria infections than hypocotyls, or a higher proportion of cotyledon cells could recover from bacterial infections and express the transferred gene than hypocotyl cells. Furthermore, no GUS staining was detected in roots of seedlings at all stages (Fig. 2). In 7-day-old seedlings, only with root tips removed did *A. tumefaciens* transiently transform 13.3% of seedling samples (Table 1), which supports the presence of wound sites in plants during *A. tumefaciens* infections is important.

**Table 1:** Transient transformation rates of *A. tumefaciens* EHA105 strain harboring the pBISN1 plasmid in ice plant seedlings and distribution (%) of GUS staining in ice plant seedlings of different ages

Ages	Plant types	Transient transformation rates^a^	% of tissues showing GUS activities^b^	
3-day-old	Intact seedling	66.7 ± 2.8*	Cotyledon	100.0 ± 2.8^#^
			Hypocotyl	14.3 ± 0.4
	Root tip removed	85.0 ± 1.1*	Cotyledon	88.9 ± 0.9^#^
			Hypocotyl	22.2 ± 0.2
5-day-old	Intact seedling	48.3 ± 3.8*	Cotyledon	63.4 ± 1.3^#^
			Hypocotyl	52.1 ± 2.5
	Root tip removed	80.0 ± 2.0*	Cotyledon	98.2 ± 2.0^#^
			Hypocotyl	25.7 ± 0.3
7-day-old	Intact seedling	0.0 ± 0.0	0.0 ± 0.0	
	Root tip removed	13.3 ± 2.8	Cotyledon	0.0 ± 0.0^#^
			Hypocotyl	100.0 ± 2.8

^a^Number of transiently transformed seedlings/total number of seedlings × 100%. The 60–80 ice plant seedlings were infected with *A. tumefaciens* strain for each independent transformation assay. Data are mean ± SD (standard deviation) from at least three independent experiments

^b^Number of GUS stained tissues/number of transiently transformed seedlings × 100%

* P < 0.05 comparing 3-, or 5-day-old infected seedlings and 7-day-old infected seedlings of the same ice plant type based on pairwise Student *t*-test

^#^P < 0.05 comparing cotyledon and hypocotyl tissues of the same age and type ice plants based on pairwise Student *t*-test

**Fig. 3:**
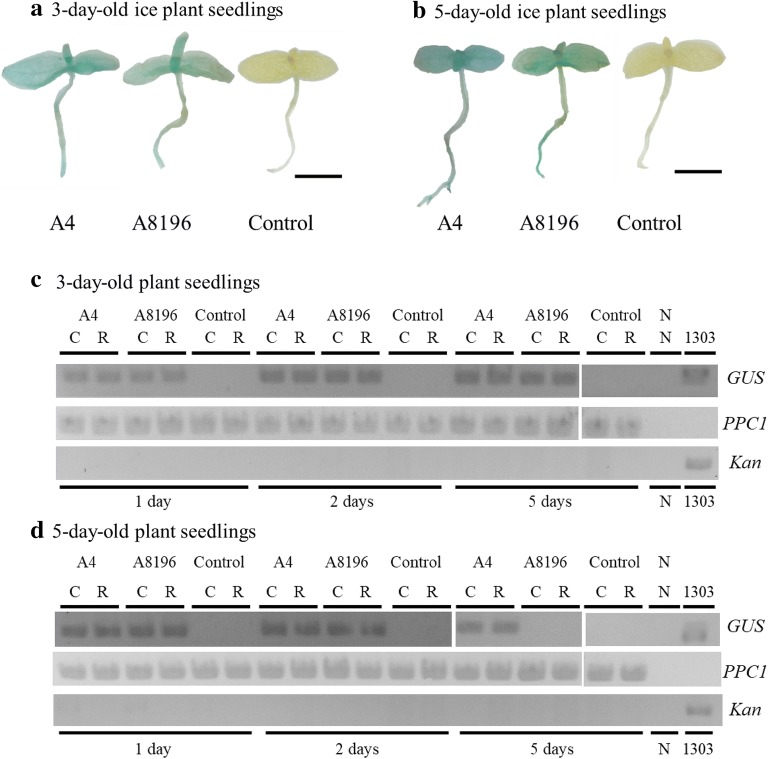
GUS staining results and genomic DNA PCR results of ice plant seedlings infected with *A. rhizogenes* A4 or A8196 strain harboring the pCAMBIA1303 binary vector. **a**, **b** Representative GUS staining results of 3- and 5-day-old seedlings infected with *A. rhizogenes* A4 or A8196 at 2 days after infection and uninfected (control) seedlings. Bar = 5 mm. **c**, **d** Genomic DNA was isolated from cotyledons (C) and roots (R) of 3- and 5-day-old infected and uninfected (control) seedlings. Distilled water was a negative control. The pCAMBIA1303 (1303) plasmid was used as a positive control for the PCR amplifications with the *GUS* reporter gene and the kanamycin resistance gene (*Kan*). Primers PPC1 3′–5 and PPC1 3′–3 were used to amplify the endogenous *PPC1* gene; primers GUS1 and GUS2 were used to amplify *GUS* reporter gene (*GUS*); primers Kan-F and Kan-R were used to amplify kanamycin resistance gene (*Kan*)

Under our infection procedures, *A. tumefaciens* was able to transform shoot but not root tissues of seedlings. To establish a transformation system in ice plant roots, we infected 3-, 5-, and 7-day-old seedlings with two types of *A. rhizogenes*, A4 and A8196, with a *GUS* reporter gene in the binary vector pCAMBIA1303. The transient transformation efficiency of seedlings was determined at 1, 2, 5, or 7 days after 2-day infection. The highest transient transformation efficiency, 100%, was with 3- and 5-day-old seedlings infected with both *A. rhizogenes* strains at 1 day after bacteria infection (Table 2). Among all tested infection conditions, 7-day-old seedlings showed 0.0% transient transformation rate (Additional file 4: Table S3). The 7-day-old seedlings might be less susceptible to *Agrobacterium* infection, which is consistent with transient transformation results obtained with *A. tumefaciens* (Table 1). Transient transformation rates with *A. rhizogenes* A4 and A8196 infection in 3-day-old seedlings remained at 92% to 100% even 5 days after infection (Table 2). Transformation rates were higher with both cotyledons and roots of 3-day-old seedlings than hypocotyls, which suggest that *A. rhizogenes* A4 and A8196 may transiently transform not only roots but also cotyledons. At 7 days after infection in 3-day-old seedlings, the transient transformation rates with *A. rhizogenes* A4 and A8196 infection both decreased significantly (Table 2). Similar results were obtained with 5-day-old seedlings in that transient transformation rates with *A. rhizogenes* A4 and A8196 were both reduced significantly at 5 days after infection (Table 2). Because the *GUS* reporter gene in the binary vector pCAMBIA1303 lacks the intron fragment, it is possible that GUS staining may result from the leaky expressions of bacterium. The representative GUS staining results showed that GUS expressions were detected in whole roots and cotyledon tissues of seedlings rather than patchy occurrence of staining caused by bacterium (Fig. 3a, b). Genomic DNA PCR results confirmed that *GUS* gene fragments were only detected in successfully transformed roots and cotyledons tissues of seedlings (Fig. 3c, d). In order to determine if the PCR amplifications of the *GUS* gene fragment might come from bacteria DNA contaminations in the genomic DNA isolations, the kanamycin resistance gene (*Kan*) located outside the T-DNA region of the pCAMBIA1303 vector was also examined by genomic DNA PCR. The genomic DNA PCR results showed that no *Kan* gene fragment from the pCAMBIA1303 vector was detected in isolated genomic DNA samples (Fig. 3c, d). In summary, these results in Tables 1 and 2 reveal that when infecting younger plant tissues, that is, 3-day-old seedlings, with *A. tumefaciens* or *A. rhizogenes* strains, GUS expression periods were longer, which suggests that younger plant tissues may be easier to be infected with *Agrobacterium* and/or younger plant tissues may more easily recover from bacteria infection and express transgenes.

**Table 2:** Transient transformation rates of *A. rhizogenes* A4 or A8196 strain harboring the pCAMBIA1303 plasmid in ice plant seedlings and distributions (%) of GUS staining in different tissues of 3- and 5-day-old ice plant seedlings

Ages of seedlings	*A. rhizogenes* strains	Days after 2-day coincubation periods	Transient transformation rates^a^	% of tissues showing GUS activities^b^	
3-day-old with root tip removed	A4	1	100.0 ± 0.0*	Cotyledon	100.0 ± 0.0^#^
				Hypocotyl	0.0 ± 0.0
				Root	95.0 ± 0.9^#^
	A8196	1	100.0 ± 0.0*	Cotyledon	100.0 ± 0.0^#^
				Hypocotyl	0.0 ± 0.0
				Root	100.0 ± 0.0^#^
	A4	2	96.7 ± 1.1*	Cotyledon	96.7 ± 0.7^#^
				Hypocotyl	6.3 ± 0.1
				Root	95.3 ± 0.1^#^
	A8196	2	100.0 ± 0.0*	Cotyledon	100.0 ± 0.0^#^
				Hypocotyl	5.6 ± 0.0
				Root	100.0 ± 0.0^#^
	A4	5	92.9 ± 2.3*	Cotyledon	83.3 ± 0.5^#^
				Hypocotyl	1.9 ± 0.6
				Root	85.2 ± 0.5^#^
	A8196	5	100.0 ± 0.0*	Cotyledon	100.0 ± 0.0^#^
				Hypocotyl	0.0 ± 0.0
				Root	100.0 ± 0.0^#^
	A4	7	33.3 ± 1.1	Cotyledon	33.3 ± 1.1^#^
				Hypocotyl	0.0 ± 0.0
				Root	31.4 ± 1.0^#^
	A8196	7	0.0 ± 0.0	Cotyledon	0.0 ± 0.0
				Hypocotyl	0.0 ± 0.0
				Root	0.0 ± 0.0
5-day-old with root tip removed	A4	1	100.0 ± 0.0*	Cotyledon	66.7 ± 1.1^#^
				Hypocotyl	0.0 ± 0.0
				Root	55.0 ± 0.9^#^
	A8196	1	100.0 ± 0.0*	Cotyledon	45.0 ± 1.2^#^
				Hypocotyl	0.0 ± 0.0
				Root	47.5 ± 1.2^#^
	A4	2	97.5 ± 0.6*	Cotyledon	65.0 ± 1.0^#^
				Hypocotyl	0.0 ± 0.0
				Root	55.0 ± 0.9^#^
	A8196	2	100.0 ± 0.0*	Cotyledon	50.0 ± 1.3^#^
				Hypocotyl	2.4 ± 0.1
				Root	47.6 ± 1.2^#^
	A4	5	47.2 ± 1.2	Cotyledon	31.5 ± 1.0^#^
				Hypocotyl	0.0 ± 0.0
				Root	29.6 ± 0.9^#^
	A8196	5	0.0 ± 0.0	Cotyledon	0.0 ± 0.0
				Hypocotyl	0.0 ± 0.0
				Root	0.0 ± 0.0
	A4	7	45.0 ± 0.0	Cotyledon	28.3 ± 0.9^#^
				Hypocotyl	0.0 ± 0.0
				Root	30.0 ± 0.9^#^
	A8196	7	0.0 ± 0.0	Cotyledon	0.0 ± 0.0
				Hypocotyl	0.0 ± 0.0
				Root	0.0 ± 0.0

^a^Number of transiently transformed seedlings/total number of seedlings × 100%. The 60 to 80 ice plant seedlings were infected with *A. rhizogenes* strain for each independent transformation assay. Data are mean ± SD (standard deviation) from at least 3 independent experiments

^b^Number of GUS stained tissues/number of transiently transformed seedlings × 100%

* P < 0.05 comparing 1-, 2-, or 5-day after infection and 7-day after infection of the same ice plant type under the same bacteria strain infection condition based on pairwise Student *t*-test

^#^P < 0.05 comparing cotyledon, or root tissues and hypocotyl tissues of the same type ice plants under the same bacteria infection condition based on pairwise Student *t*-test

### Establishing transgenic ice plant roots expressing GUS via *A. rhizogenes* infection

We have shown that using ice plant seedlings is a feasible system to test transient transformation efficiency, but seedlings were difficult to resume growth after infection. Table 2 showed ice plant roots were readily infected by *A. rhizogenes* and were able to maintain T-DNA expression up to 7 days. In an attempt to generate transgenic roots, we syringe-infected 5- to 6-week-old pot-grown plants with *A. rhizogenes*. Two *A. rhizogenes* strains A8196 and NCPPB 1855, harboring the binary vector pCAMBIA1303 were used to infect plants. Infected plants were removed from soil 1 week after infection and were grown hydroponically in modified Johnson’s nutrient solution.

**Fig. 4:**
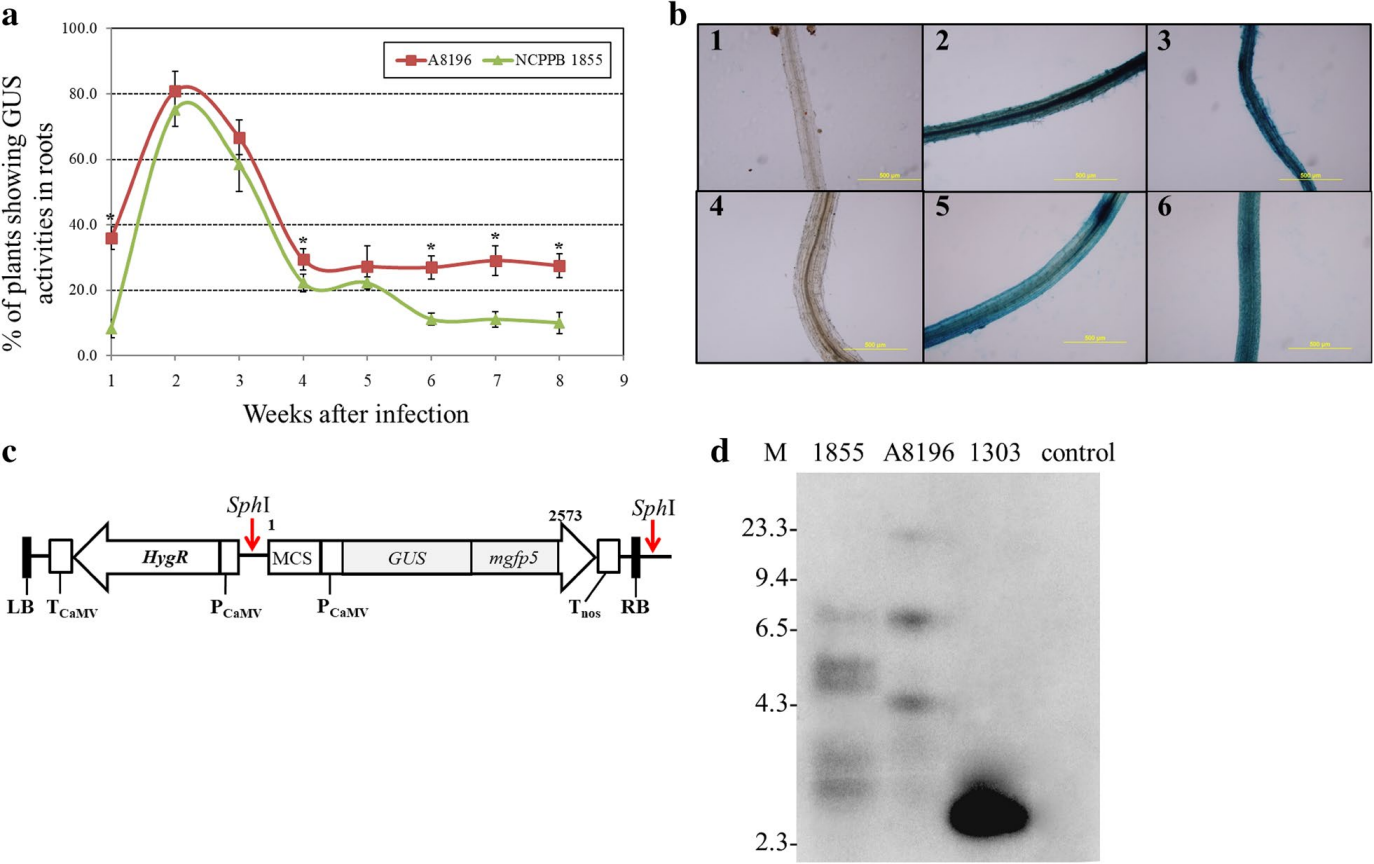
Transformation efficiency and Southern blotting results of hydroponically grown ice plants after infection with the *A. rhizogenes* A8196 or NCPPB 1855 strain harboring the pCAMBIA1303 binary vector. **a** Percentage of plants showing GUS activity in hydroponically grown plant roots on infection with *A. rhizogenes* A8196 or NCPPB 1855 from 1 to 8 weeks after infection. About 16–20 ice plants were infected with either *A. rhizogenes* strain for each independent transformation assay. Data are mean ± SD from at least three independent experiments. *P < 0.05 comparing *A. rhizogenes* A8196 infected plants and *A. rhizogenes* NCPPB 1855 infected plants under the same week after infection based on pairwise Student *t*-test. **b** Representative GUS staining of ice plant roots after infiltration with mock control (b1, b4), infection with *A. rhizogenes* A8196 (b2, b5) or NCPPB 1855 (b3, b6). Mock control or bacteria-infected root fragments from plants 1 week after treatments are shown in b1 to b3 and 8 weeks after treatments in b4 to b6. Bar = 500 μm. **c** T-DNA regions of the binary vector pCAMBIA1303 showing the positions of *Sph*I site. **d** Genomic Southern blot analysis of T-DNA integration 8 weeks after infiltration. Genomic DNA (30 μg/lane) was digested with *Sph*I and hybridized with ^32^P-labeled GUS probe. M: λDNA *Hin*dIII-digested marker; 1855: NCPPB 1855-infected roots; 8196: A8196-infected roots; 1303: gel-purified *Sph*I-digested fragment of pCAMBIA1303; control: mock control roots

**Fig. 5:**
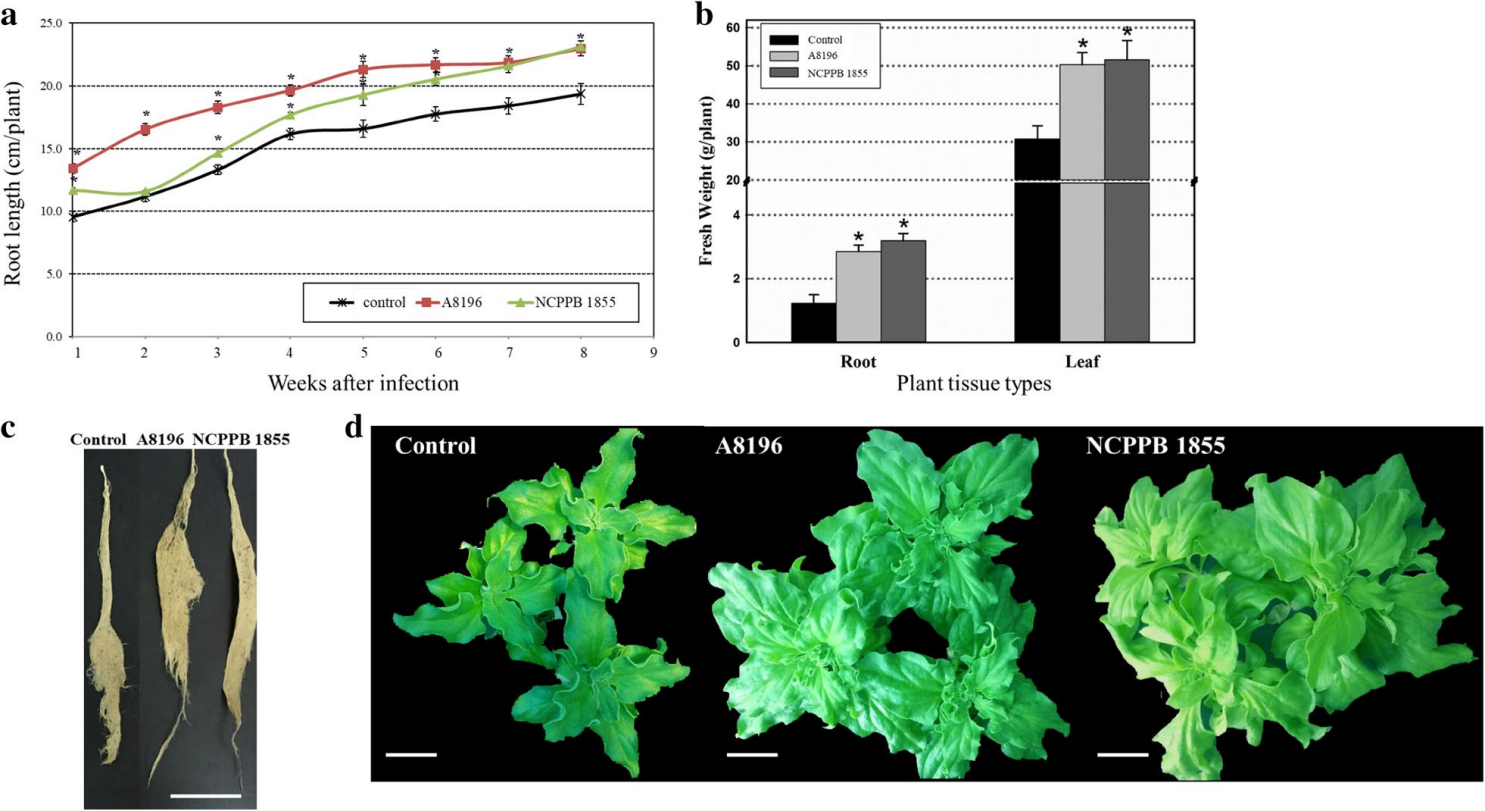
The growth of hydroponically grown ice plants after infection by *A. rhizogenes* strains. **a** Root length of mock control, *A. rhizogenes* A8196- or NCPPB 1855-infected plants was measured every week for 8 weeks. *P < 0.05 comparing *A. rhizogenes*-infected plants and mock control based on pairwise Student *t*-test. **b** Fresh weight of roots and aerial parts (leaf) of infected plants measured after 8-week infection. About 16–20 ice plants were infected with each *A. rhizogenes* strain for each independent transformation assay. Average values of the fresh weights from at least three independent experiments. Error bars were calculated using Excel STDEVP function. *P < 0.05 comparing *A. rhizogenes*-infected plants and mock control based on pairwise Student *t*-test. The representative root (**c**) and aerial tissues (**d**) of plants infiltrated with mock control, *A. rhizogenes* A8196 or NCPPB 1855 (from left to right) after 8-week treatments. Bar = 50 μm. NaCl solution used to wash and resuspend bacteria was the mock control

At 1 week after infection, plants infected with *A. rhizogenes* A8196 showed higher percentage of GUS activities (35.9%) in roots than plants infected with NCPPB 1855 (Fig. 4a). These data indicated that *A. rhizogenes* A8196 had higher transient transformation efficiency. Representative mock control ice plants and plants infected with *A. rhizogenes* A8196 and NCPPB 1855 showing GUS activity in roots at 1 week after infection, are shown in the Fig. 4b—1, 2, 3, respectively. At 2 weeks after infection, the transformation efficiency on infection with the two *A. rhizogenes* strains continued to increase to 75–81% (Fig. 4a). The transformation efficiencies on infection with the two *A. rhizogenes* strains all gradually decreased at 3 weeks after infection (Fig. 4a). At 4 weeks after infection, the transformation rates of *A. rhizogenes* strains A8196 and NCPPB 1855 was 29.4% ± 3.2% and 22.2% ± 2.6%, respectively (Fig. 4a). Results shown in the Fig. 4a demonstrated that transformation rates at 4 weeks after infection with two *A. rhizogenes* strains were 2.8- to 3.4-fold lower than transformation rates at 2 weeks after infection. Transformation efficiency remained relatively higher on infection with *A. rhizogenes* A8196 than the NCPPB 1855 strain (Fig. 4a). These results suggest that ice plant may be more susceptible to infection with *A. rhizogenes* A8196. The percentage of plants with GUS activity in roots peaked at 2 weeks after infection and remained with no significant change since 4 weeks after infection, so the decrease in the transformation efficiency at 3 weeks after infection might be due to the lack of stable T-DNA integration or lack of T-DNA-encoded gene expression in the transformed plants. Representative mock control plants and plants infected with *A. rhizogenes* A8196 and NCPPB 1855 showing GUS activities in roots at 8 weeks are shown in Fig. 4b—4, 5, 6, respectively.

To examine the T-DNA integration in infected roots, Southern blotting was performed in roots infected with *A. rhizogenes* A8196 and NCPPB 1855 at 8 weeks after infection. There are two *Sph*I sites in the pCAMBIA1303: one is around the multiple cloning sites, and another one is just outside of T-DNA right border (Fig. 4c). Genomic DNA was digested with *Sph*I and detected by a ^32^P-labeled *GUS* probe. At least 3 and 5 hybridizing fragments were detected in roots infected with A8196 and NCPPB 1855, respectively. The sizes of all the *Sph*I-digested fragments were larger than the *GUS*-containing fragment of *Sph*I-digested pCAMBIA1303 plasmid, indicating multiple independent insertion events may have occurred in both infection treatments (Fig. 4d). In order to examine if bacteria DNA might exist in our genomic DNA isolation from ice plants, genomic DNA PCR with the *Kan* gene was also performed. Genomic DNA PCR results showed that no *Kan* gene fragment was amplified from genomic DNA isolated from the *A. rhizogenes* A8196 and NCPPB 1855-infected ice plants root tissues (Additional file 5: Figure S2A). GUS protein was detected in roots infected with the two *A. rhizogenes* strains but not mock control plants (Additional file 5: Figure S2B). In addition, no GUS protein was detected in leaf tissues of plants infected with the *A. rhizogenes* strains at 8 weeks after infection (Additional file 5: Figure S2C). These immunoblot results were consistent with observations of no GUS activity detected in leaf tissues of transformed plants infected with the two *A. rhizogenes* strains (Additional file 6: Figure S3).

The fresh weight and length of roots of plants infected with *A. rhizogenes* strains were examined to determine growth effects of transgenic roots on hydroponically grown plants. The root lengths of infected plants and mock control plants were measured every week from 7 days after infections (Fig. 5a). The root lengths of the ice plants infected with *A. rhizogenes* A8196 were the longest as compared with the other infected and mock control plants from 1 week after infection (Fig. 5a). The root lengths of *A. rhizogenes* NCPPB 1855-infected plants started to catch up at week 3 and became indistinguishable at week 7 from A8196-infected plants (Fig. 5a). The root lengths of *A. rhizogenes* A8196- and NCPPB 1855-infected plants were 20% longer than the mock control plants at 8 weeks after infection. The root fresh weights of plants infected with A8196 and NCPPB 1855 increased 2.3- to 2.6-fold compared to mock control plants at the end of 8-week period (Fig. 5b, c). Because of the improved root growth of *A. rhizogenes*-infected plants, the fresh weights of aerial parts of infected plants also increased. The fresh weights of aerial parts of plants infected with two *A. rhizogenes* strains increased 1.7-fold as compared with mock control plants (Fig. 5b, d). The results showed two strains of *A. rhizogenes* efficiently infect the roots of ice plant and promote growth of the whole plant.

In summary, we obtained the highest transient transformation efficiency with 5-day-old ice plant callus when co-incubated with an *A. tumefaciens* at 2.5 × 10^9^ cfu mL^−1^ for 2 days. We also successfully used the *A. tumefaciens* and *A. rhizogenes* to transiently transform ice plant seedlings and obtained 66–85% and 33–100% transient transformation rates in 3-day-old seedlings, respectively. Finally, pot-grown juvenile plants were infected with *A. rhizogenes*, and then were grown hydroponically to obtain transformed roots expressing GUS reporter genes. More than 27% of plants which were infected with *A. rhizogenes* A8196 had transformed roots for 8 consecutive weeks.

## Discussion

Global climate change has caused rising temperatures and decreasing rainfall, which increases agricultural irrigation needs and vapor transpiration rates, and in turn accelerates the risk of soil salinity and jeopardizes plant growth and crop yields. The ice plant is a halophyte that can tolerate high levels of Na^+^ equivalent to that in seawater during plant growth. It is an important model species for researching plant salinity responses and the inducible CAM pathways (Bohnert and Cushman 2000; Cushman and Borland 2002). However, the lack of an easy and efficient transformation system has impeded functional studies of stress-related genes in ice plants. In this study, we used two types of *Agrobacterium* strains, *A. tumefaciens* and *A. rhizogenes*, to establish transient transformation methods with three kinds of ice plant materials, callus, seedlings and pot-grown plants. Among all the tested *Agrobacterium* strains, *A. tumefaciens* EHA105 and *A. rhizogenes* A4 strains yielded the best transient transformation rates with 3-day-old seedlings with root tip removed. The 5-day-old callus showed the highest transient transformation efficiency. Additionally, the *A. rhizogenes* A8196 strain produced the highest percentage of ice plants with transformed roots.

The plant materials frequently used to infect with *Agrobacterium* and establish transformation systems are mature embryos, immature embryos, or callus from explants of leaves, roots, cotyledons, hypocotyls, and other tissues. These plant cells tend to be vigorously dividing or about to divide and can regenerate easily (Mannan et al. 2009; Anami et al. 2013). The proteins involved in DNA replication and repair machinery are highly expressed in these actively dividing cells and play key roles in establishing successful transformation methods (Gelvin 2017). Therefore, we used ice plant callus to be infected with *A. tumefaciens* and establish transformation protocols. The younger plant callus, 5- and 7-day-old callus, yielded higher transient transformation rates than 14-day-old callus (Fig. 1b). Ishimaru (1999) reported that no transformed callus was obtained from explants of 2-week-old leaf, cotyledon, and callus tissues. In this study, we have successfully transiently transformed 5-day-old callus with 3% transformation rates. These data suggest that younger plant cells may be more susceptible to *Agrobacterium* infection than older cells and the ability to vigorously divide or regenerate in plant materials is important in successful transformation. Similar results were obtained with other plant species, including rice, maize, tomato, and loblolly pine (*Pinus taeda* L.), showing younger plant cells more susceptible to *Agrobacterium* infection than older cells (Hiei et al. 1997; Tang 2001; Cheng et al. 2004; Qiu et al. 2007). In this study, more than 92% of 3-day-old ice plant seedlings showed transgene expressions in root and cotyledon tissues after infected with *A. rhizogenes.* Compared to previous studies (Andolfatto et al. 1994; Konieczny et al. 2011), relatively higher transient transformation efficiencies were obtained in this study might be due to different age of ice plant seedlings were used. We have utilized much younger seedlings, 3-day-old seedlings, to establish transformation system instead of 14-day-old seedlings which were used in studies of Andolfatto et al. (1994) and Konieczny et al. (2011). These data also demonstrate that careful selection of the type and quality of the plant materials used for transformation is crucial for establishing effective transformation methods.

Another vital factor that may contribute to the success of transient and stable transformation assay is the concentration of bacteria culture. Konieczny et al. (2011) reported that optimal density of *A. rhizogenes* culture for ice plant seedlings transformation was OD_600_ = 2 (around 2 × 10^9^ cfu mL^−1^) and higher bacteria concentration caused significant decreases in transformation efficiencies. In this study, we used the bacteria concentration 2.5 × 10^9^ cfu mL^−1^ to infect ice plant callus for 48 h, and showed the highest transient transformation efficiency at 24 h after infection (Fig. 1a). Furthermore, the seedlings were infected with 2.5 × 10^9^ cfu mL^−1^ *A. tumefaciens* or 2 × 10^9^ cfu mL^−1^ *A. rhizogenes* to establish successful transient transformation systems. These data and previous studies both suggest that the best bacteria concentration used to infect plant cells ranges from 10^8^ to 10^9^ cfu mL^−1^ (Tepfer 1984; Andolfatto et al. 1994; Ishimaru 1999; Hiei et al. 1997; Tang 2001; Cheng et al. 2004; Konieczny et al. 2011). A too-high bacteria concentration may cause too much plant tissue damage and cell death from bacteria infection and a too-low concentration may not produce enough T-DNA and Vir proteins for effective transformation.

A previous study has used three *A. rhizogenes* strains A4, C58C1 (pRi8196), and ARqua1 to transform 2-week-old ice plant seedlings and produce transgenic roots with 20% transformation rates (Andolfatto et al. 1994). Similarly, Konieczny et al. (2011) utilized the *A. rhizogenes* strain ARqua1 to infect 7 and 14-day-old ice plant seedlings to obtain transgenic roots with 6% and 20% transformation efficiencies, respectively. The transformed roots showed the hairy root phenotype and were grown in liquid MS media without exogenous plant hormones for more than 2 years. In addition, the transformed callus was induced and obtained from these transgenic hairy roots which were cultured in the solidified MS media with cytokinin and auxin for at least 4–6 weeks (Konieczny et al. 2011). In our study, we have first initiated and maintained large amounts ice plant callus in CIM media. Next, we have effectively used the *A. tumefaciens* strain to infect ice plant callus; expressed transgene in these ice plant callus within 4 weeks using our established protocols. This experimental strategy has provided us with shorter time period to establish transgenic ice plant callus and more materials to study various gene functions in ice plant callus.

Another vital factor that may contribute high transient transformation efficiencies might be full induction of *vir* gene expressions in *Agrobacterium*. Transient transformation efficiencies of ice plant seedlings were 1.3- to 1.7-fold higher when root tips were removed in comparison to intact seedlings (Table 1). These data further support that the existence of wounded sites in plants may help with *Agrobacterium* infection by producing a phenolic compound to induce *vir* gene expression and making plant cells vigorously divide to be competent for transformation. In addition, we have added 200 μM acetosyringone (AS) in our bacteria culture to fully induce *vir* gene expressions in *Agrobacterium*. In previous studies (Andolfatto et al. 1994; Ishimaru 1999; Konieczny et al. 2011), *Agrobacterium* strains were only cultured in rich media without additions of AS. In this study, *Agrobacterium* was specifically cultured in acidic minimal medium (AB-MES media), which contained glucose and low phosphate, and 200 μM AS to maximize *vir* gene expression in bacteria and may therefore result in successful and more effective transformation in ice plant callus and seedlings.

Although efficient transient transformation system of aseptically grown ice plant seedlings was successfully established in this study, no stable transformed roots were acquired from these seedlings. Andolfatto et al. (1994) and Konieczny et al. (2011) both reported labor-intensive and time-consuming transformation and tissue culture protocols to establish ice plants containing transgenic roots with relatively low transformation rates, 6–20%. In order to circumvent the long and grueling tissue culture system of whole-plant regeneration from ice plant callus or seedlings, pot-grown ice plants were first syringe-injected with *A. rhizogenes* A8196 or NCPPB 1855 bacteria cultures and then grown hydroponically to induce transgenic root formations; more than 35% of plants contained transgenic roots at 1 week after infected with *A. rhizogenes* A8196. Two weeks after infections, more than 75% of plants had transgenic roots after infections. These infected plants were continuously grown hydroponically for transgene expressions in root tissues without generating whole new transgenic plants.

Among the two tested *A. rhizogenes* strains, *A. rhizogenes* A8196 had higher transformation efficiency than the *A. rhizogenes* NCPPB 1855 in hydroponically grown ice plants (Fig. 4a), so *A. rhizogenes* A8196 may be more suitable to establish composite plant systems in ice plants than the other strain. However, GUS activities remained higher than 97% in both *A. rhizogenes* A4- and A8196-infected seedlings at 2 days after infection (Table 2), indicating ice plant seedlings were both highly susceptible to agropine-type A4 and mannopine-type A8196 infection. These different results may be caused by different ages of plant materials and/or different infection methods used to examine transformation efficiency.

The *A. rhizogenes* causes hairy root productions due to the transfer, integration, and expression of the T-DNA from the Ri plasmid (Chilton et al. 1982; White et al. 1982). *A. rhizogenes*-mediated transformation has been used to study gene functions, produce transgenic roots and/or plants, produce secondary metabolite, and study plant–pathogen interactions (Christey 2001; Veena and Taylor 2007; Ono and Tian 2011). Genes located in the T-DNA regions of the Ri plasmid are involved in root induction and development (*rol* genes), opine biosynthesis, auxin biosynthesis and unknown functions (Veena and Taylor 2007; Georgiev et al. 2012). Previous studies have suggested that transformation by *A. rhizogenes* may cause changes in concentration and metabolism of different plant hormones in host tissues of several plant species, including ice plants (Nilsson et al. 1993; Lambert et al. 1998; Christey 2001; Konieczny et al. 2011). In our study, we have observed that these *A. rhizogenes*-infected ice plants showed better growth not only in roots but also in aerial parts of ice plants. The improved vegetative growth in *A. rhizogenes*-infected ice plants might be due to change of plant hormone amounts in host tissues. The effects of *A. rhizogenes* transformation on hormone amounts and metabolism in ice plant tissues await further investigations.

## Conclusions

Efficient *Agrobacterium*-mediated transformation protocols were established in callus, seedlings, and roots of halophyte *M. crystallinum*. The successfully established transformation systems by *A. tumefaciens* and *A. rhizogenes* will provide the plant research community with more effective tools to study gene functions in ice plants.

## Additional files

**Additional file 1: Table S1.** Bacterial strains and plasmids used in this study.

**Additional file 2: Table S2.** List of oligonucleotide primers used in this study.

**Additional file 3: Figure S1.** The establishment of transgenic ice plant callus expressing the yellow fluorescent protein (YFP). (A) Representative untransformed ice plant callus grown on CIM with (1) 0 mg L^−1^ and (2) 25 mg L^−1^ glufosinate ammonium. Bar = 1 cm. (A-3) Fresh weight of untransformed ice plant callus grown on CIM with 0, 25, 50, 75, and 100 mg L^−1^ glufosinate ammonium (GLA). ΔFW: increased fresh weight mass. Average values of the increased fresh weight from at least three independent experiments. *P < 0.05 comparing with and without GLA by pairwise Student *t*-test. (B) Fluorescent observation of *YFP*-transformed callus-derived cells. (B1-3) Untransformed callus-derived cells; (B4-6) YFP-transformed callus-derived cells; (B1, B4) Bright field images; (B2, B5) Confocal microscopy images with the YFP filter; (B3, B6) merged images. Bar = 20 μm. (C) Genomic DNA PCR results of *YFP* (upper panel) and phosphoenolpyruvate carboxylase (*PPC1*) (lower panel) in plant callus. 1, 2: independent transformed plant callus lines; NC: untransformed callus as a negative control; arrow: predicted length of PCR product.

**Additional file 4: Table S3.** Transient transformation rates of *A. rhizogenes* A4 or A8196 strain harboring the pCAMBIA1303 plasmid in 7-day-old ice plant seedlings and distributions (%) of GUS staining in different tissues of 7-day-old ice plant seedlings.

**Additional file 5: Figure S2.** Genomic DNA PCR results and immunoblot results of hydroponically grown ice plants after infection with the *A. rhizogenes* A8196 or NCPPB 1855 strain harboring the pCAMBIA1303 binary vector. (A) Genomic DNA was isolated from roots of *A. rhizogenes* A8196- or NCPPB 1855-infected plants infected and uninfected (mock control) plants. Distilled water was a negative control (N) in genomic DNA PCR. The pCAMBIA1303 (1303) plasmid was used as a positive control for the PCR reactions with primers of the *GUS* gene or the kanamycin resistance gene (*Kan*). (B) Immunoblot analysis of GUS protein and Coomassie blue staining of proteins extracted from root tissues of mock control and *A. rhizogenes* A8196- or NCPPB 1855-infected plants shown in the upper and lower panels, respectively. The antibody against ß-glucuronidase (GUS protein) was used for immunoblot analysis. (C) Immunoblot analysis of GUS protein and Coomassie blue staining of proteins extracted from roots or leaves of mock control and infected plants. The position of GUS is indicated. NaCl solution used to wash and resuspend bacteria was the mock control.

**Additional file 6: Figure S3.** Representative GUS staining results of leaves from hydroponically grown ice plants after infected with the *A. rhizogenes* A8196 or NCPPB 1855 strains. Leaves of ice plants after infiltration with mock control (Panel A, D), infection with *A. rhizogenes* A8196 (Panel B, E) or NCPPB 1855 (Panel C, F). Mock control or bacteria infected leaves from plants 8 weeks after treatments are shown in panel A to C. Leaves after GUS staining are shown in panel D to F. Bar = 1 cm.
