# Supplementary material for: Effective Agrobacterium-mediated transformation protocols for callus and roots of halophyte ice plant (Mesembryanthemum crystallinum)
Source: Bot Stud. 2019 Jan 7;60:1. doi: 10.1186/s40529-018-0249-3 (PMC6323063; doi:10.1186/s40529-018-0249-3)
Supplement: Supplementary file 1 — Additional file 1: Table S1. Bacterial strains and plasmids used in this study. [file 40529_2018_249_MOESM1_ESM.doc]

**Table S1. Bacterial strains and plasmids used in this study**

| Strains  *Agrobacterium tumefaciens* | Relevant characteristics | References/sources |
| --- | --- | --- |
| A136 | Rm^R^, C58 cured of its Ti plasmid pTiC58 | Watson et al. 1975 |
| EHA105 | Rm^R^, A136 strain containing a disarmed agropine-type Ti plasmid pEHA105 | Hood et al. 1986 |
| *Agrobacterium rhizogenes* |  |  |
| A4 | Rm^R^, A136 strain containing a virulent Ri plasmid pRiA4 | Tepfer, 1984 |
| A8196 | wild-type virulent strain containing a virulent Ri plasmid pRi8196 | Chilton et al. 1982 |
| NCPPB 1855 | wild-type virulent strain containing a virulent Ri plasmid pRi1855 | Young et al. 2001 |
| *Escherichia coli* |  |  |
| DH5α | Host for DNA cloning | Hanahan, 1983 |
| DH10B | Host for DNA cloning | Invitrogen, Carlsbad, CA, USA |
| Plasmids |  |  |
| pBISN1 | Km^R^, a binary vector expressing *gusA*-intron driven by a super promoter | Narasimhulu et al. 1996 |
| pBA002 | Sp^R^, a binary vector expressing *yfp* driven by a CaMV 35S promoter | Kost et al. 1998 |
| pCAMBIA1303 | Km^R^, a binary vector expressing *gusA:mgfp5* fusion driven by a CaMV 35S promoter | Hajdukiewicz et al. 1994; Hiei et al. 1994 |

Km^R^, Rm^R^, and Sp^R^ = Resistant to kanamycin, rifampicin, and spectinomycin, respectively

**References:**

Chilton MD, Tepfer DA, Petit A, David C, Casse-Delbart F, Tempe J (1982) *Agrobacterium rhizogenes* inserts T-DNA into the genomes of the host plant root cells. Nature 295:432－434

Hajdukiewicz P, Svab Z, Maliga P (1994) The small versatile pPZP family of *Agrobacterium* binary vectors for plant transformation. Plant Mol Biol 25:989－994

Hanahan D (1983) Studies on transformation of *Escherichia coli* with plasmids. J Mol Biol 166:557－580

Hiei Y, Ohta S, Komari T, Kumashiro T (1994) Efficient transformation of rice (*Oryza sativa* L.) mediated by *Agrobacterium* and sequence analysis of the boundaries of the T-DNA. Plant J 6:271－282

Hood EE, Chilton WS, Chilton MD, Fraley RT (1986) T-DNA and opine synthetic loci in tumors incited by *Agrobacterium tumefaciens* A281 on soybean and alfalfa plants. J Bacteriol 168:1283－1290

Kost B, Spielhofer P, Chua NH (1998) A GFP-mouse talin fusion protein labels plant actin filaments *in vivo* and visualizes the actin cytoskeleton in growing pollen tubes. Plant J 16:393－401

Narasimhulu SB, Deng XB, Sarria R, Gelvin SB (1996) Early transcription of *Agrobacterium* T-DNA genes in tobacco and maize. Plant Cell 8:873－886

Tepfer D (1984) Transformation of several species of higher plants by *Agrobacterium rhizogenes*: sexual transmission of the transformed genotype and phenotype. Cell 37:959－967

Watson B, Currier TC, Gordon MP, Chilton MD, Nester EW (1975) Plasmid required for virulence of *Agrobacterium tumefaciens*. J Bacteriol 123:255－264

Young JM, Kuykendall LD, Martínez-Romero E, Kerr A, Sawada H (2001) A revision of *Rhizobium* Frank 1889, with an emended description of the genus, and the inclusion of all species of *Agrobacterium* Conn 1942 and *Allorhizobium undicola* de Lajudie *et al*. 1998 as new combinations: *Rhizobium radiobacter*, *R. rhizogenes, R. rubi, R. undicola* and *R. vitis.* Int J Syst Evol Microbiol 51:89－103
