# Supplementary material for: Effective Agrobacterium-mediated transformation protocols for callus and roots of halophyte ice plant (Mesembryanthemum crystallinum)
Source: Bot Stud. 2019 Jan 7;60:1. doi: 10.1186/s40529-018-0249-3 (PMC6323063; doi:10.1186/s40529-018-0249-3)
Supplement: Supplementary file 2 — Additional file 2: Table S2. List of oligonucleotide primers used in this study. [file 40529_2018_249_MOESM2_ESM.doc]

**Table S2. List of oligonucleotide primers used in this study**

| **Name** | **Primer sequence** |
| --- | --- |
| GUS1 | 5’-GGGCAGGCCAGCGTATCGTG-3’ |
| GUS2 | 5’-GTCCCGCTAGTGCCTTGTCCAGTT-3’ |
| YFP-F | 5’-GTGAGCAAGGGCGAGGAG-3’ |
| YFP-R | 5’-CGTCCATGCCGAGAGTGA-3’ |
| PPC13’-5 | 5’-ATCCCGACTTCAAGGTAACAGAG-3’ |
| PPC13’-3 | 5’-CGATGCCCATAAATAAACAAGAAA-3’ |
| Kan-F | 5’-ATGGCTAAAATGAGAATATCA-3’ |
| Kan-R | 5’-CTAAAACAATTCATCTAGTAA-3’ |
