# Supplementary material for: Effective Agrobacterium-mediated transformation protocols for callus and roots of halophyte ice plant (Mesembryanthemum crystallinum)
Source: Bot Stud. 2019 Jan 7;60:1. doi: 10.1186/s40529-018-0249-3 (PMC6323063; doi:10.1186/s40529-018-0249-3)
Supplement: Supplementary file 3 — Additional file 3: Figure S1. The establishment of transgenic ice plant callus expressing the yellow fluorescent protein (YFP). (A) Representative untransformed ice plant callus grown on CIM with (1) 0 mg L−1 and (2) 25 mg L−1 glufosinate ammonium. Bar = 1 cm. (A-3) Fresh weight of untransformed ice plant callus grown on CIM with 0, 25, 50, 75, and 100 mg L−1 glufosinate ammonium (GLA). ΔFW: increased fresh weight mass. Average values of the increased fresh weight from at least three independent experiments. *P < 0.05 comparing with and without GLA by pairwise Student t-test. (B) Fluorescent observation of YFP-transformed callus-derived cells. (B1-3) Untransformed callus-derived cells; (B4-6) YFP-transformed callus-derived cells; (B1, B4) Bright field images; (B2, B5) Confocal microscopy images with the YFP filter; (B3, B6) merged images. Bar = 20 μm. (C) Genomic DNA PCR results of YFP (upper panel) and phosphoenolpyruvate carboxylase (PPC1) (lower panel) in plant callus. 1, 2: independent transformed plant callus lines; NC: untransformed callus as a negative control; arrow: predicted length of PCR product. [file 40529_2018_249_MOESM3_ESM.doc]

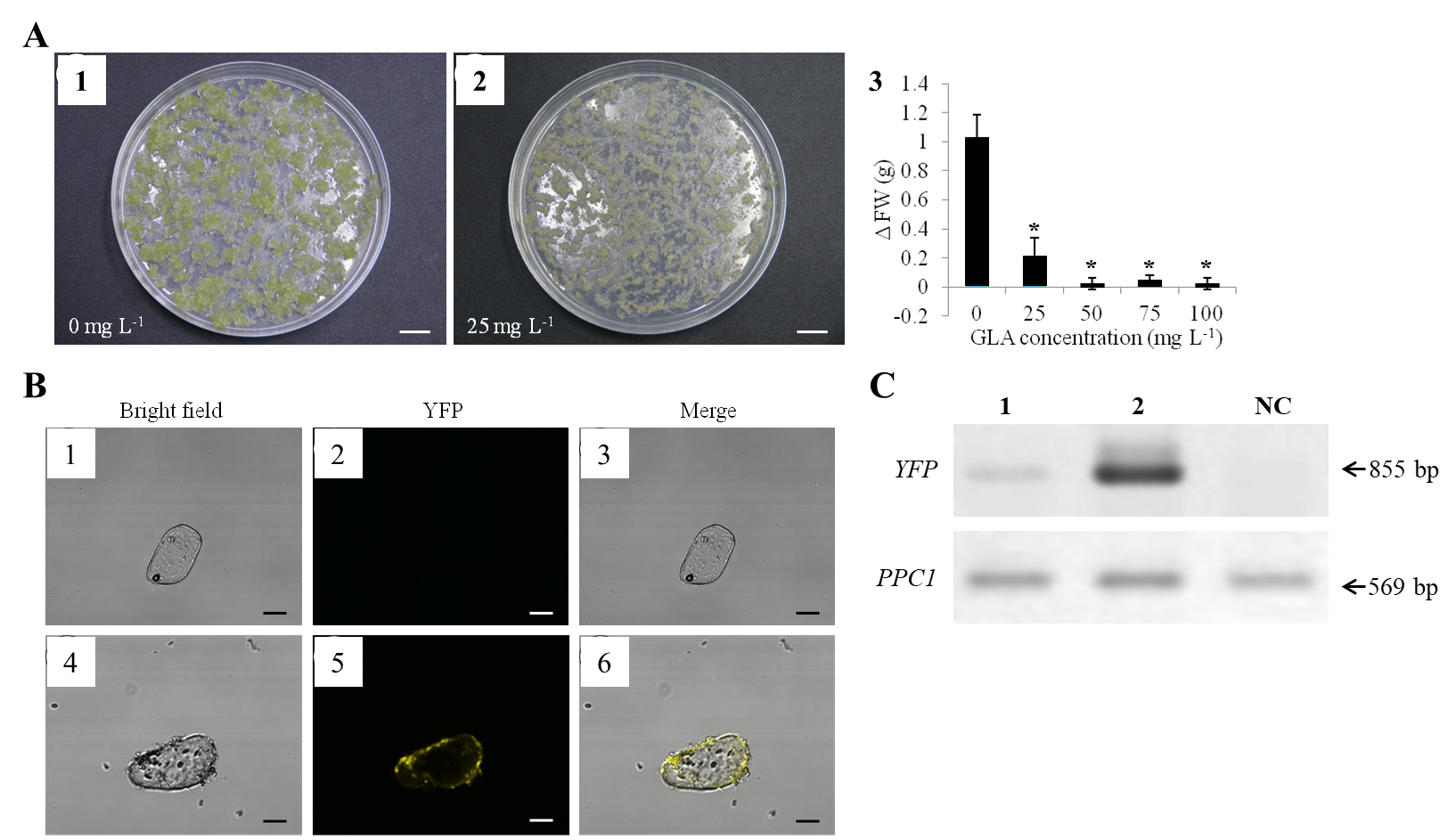


**Figure S1**. **The establishment of transgenic ice plant callus expressing the yellow fluorescent protein (YFP).** (A) Representative untransformed ice plant callus grown on CIM with (1) 0 mg L^-1^ and (2) 25 mg L^-1^ glufosinate ammonium. Bar = 1 cm. (A-3) Fresh weight of untransformed ice plant callus grown on CIM with 0, 25, 50, 75, and 100 mg L^-1^glufosinate ammonium (GLA). ΔFW: increased fresh weight mass. Average values of the increased fresh weight from at least three independent experiments. ＊ P<0.05 comparing with and without GLA by pairwise Student *t-*test. (B) Fluorescent observation of *YFP*-transformed callus-derived cells. (B1-3) Untransformed callus-derived cells; (B4-6) YFP-transformed callus-derived cells; (B1, B4) Bright field images; (B2, B5) Confocal microscopy images with the YFP filter; (B3, B6) merged images. Bar = 20 μm. (C) Genomic DNA PCR results of *YFP* (upper panel) and phosphoenolpyruvate carboxylase (*PPC1*) (lower panel) in plant callus. 1, 2: independent transformed plant callus; NC: untransformed callus as a negative control; arrow: predicted length of PCR product.
