# Supplementary material for: Effective Agrobacterium-mediated transformation protocols for callus and roots of halophyte ice plant (Mesembryanthemum crystallinum)
Source: Bot Stud. 2019 Jan 7;60:1. doi: 10.1186/s40529-018-0249-3 (PMC6323063; doi:10.1186/s40529-018-0249-3)
Supplement: Supplementary file 4 — Additional file 4: Table S3. Transient transformation rates of A. rhizogenes A4 or A8196 strain harboring the pCAMBIA1303 plasmid in 7-day-old ice plant seedlings and distributions (%) of GUS staining in different tissues of 7-day-old ice plant seedlings. [file 40529_2018_249_MOESM4_ESM.doc]

**Table S3. Transient transformation rates of *A. rhizogenes* A4 or A8196 strain harboring the pCAMBIA1303 plasmid in 7-day-old ice plant seedlings and distributions (%) of GUS staining in different tissues of 7-day-old ice plant seedlings.**

| **Age of seedlings** | ***A. rhizogenes* strains** | **Days after 2-day coincubation periods** | **Transient transformation rates^a^** | **% of tissues showing GUS activities^b^** | |
| --- | --- | --- | --- | --- | --- |
| 7-day-old with root tip removed | A4 | 1 | 0.0 ± 0.0 | Cotyledon | 0.0 ± 0.0 |
|  |  |  |  | Hypocotyl | 0.0 ± 0.0 |
|  |  |  |  | Root | 0.0 ± 0.0 |
|  | A8196 | 1 | 0.0 ± 0.0 | Cotyledon | 0.0 ± 0.0 |
|  |  |  |  | Hypocotyl | 0.0 ± 0.0 |
|  |  |  |  | Root | 0.0 ± 0.0 |
|  | A4 | 2 | 0.0 ± 0.0 | Cotyledon | 0.0 ± 0.0 |
|  |  |  |  | Hypocotyl | 0.0 ± 0.0 |
|  |  |  |  | Root | 0.0 ± 0.0 |
|  | A8196 | 2 | 0.0 ± 0.0 | Cotyledon | 0.0 ± 0.0 |
|  |  |  |  | Hypocotyl | 0.0 ± 0.0 |
|  |  |  |  | Root | 0.0 ± 0.0 |
|  | A4 | 5 | 0.0 ± 0.0 | Cotyledon | 0.0 ± 0.0 |
|  |  |  |  | Hypocotyl | 0.0 ± 0.0 |
|  |  |  |  | Root | 0.0 ± 0.0 |
|  | A8196 | 5 | 0.0 ± 0.0 | Cotyledon | 0.0 ± 0.0 |
|  |  |  |  | Hypocotyl | 0.0 ± 0.0 |
|  |  |  |  | Root | 0.0 ± 0.0 |
|  | A4 | 7 | 0.0 ± 0.0 | Cotyledon | 0.0 ± 0.0 |
|  |  |  |  | Hypocotyl | 0.0 ± 0.0 |
|  |  |  |  | Root | 0.0 ± 0.0 |
|  | A8196 | 7 | 0.0 ± 0.0 | Cotyledon | 0.0 ± 0.0 |
|  |  |  |  | Hypocotyl | 0.0 ± 0.0 |
|  |  |  |  | Root | 0.0 ± 0.0 |

^1^ Number of transiently transformed seedlings/total number of seedlings ×100%. The 60 to 80 ice plant seedlings were infected with *A. rhizogenes* strain for each independent transformation assay. Data are mean±SD (standard deviation) from at least 3 independent experiments.

^2^ Number of GUS stained tissues/number of transiently transformed seedlings ×100%
