# Supplementary material for: Effective Agrobacterium-mediated transformation protocols for callus and roots of halophyte ice plant (Mesembryanthemum crystallinum)
Source: Bot Stud. 2019 Jan 7;60:1. doi: 10.1186/s40529-018-0249-3 (PMC6323063; doi:10.1186/s40529-018-0249-3)
Supplement: Supplementary file 5 — Additional file 5: Figure S2. Genomic DNA PCR results and immunoblot results of hydroponically grown ice plants after infection with the A. rhizogenes A8196 or NCPPB 1855 strain harboring the pCAMBIA1303 binary vector. (A) Genomic DNA was isolated from roots of A. rhizogenes A8196- or NCPPB 1855-infected plants infected and uninfected (mock control) plants. Distilled water was a negative control (N) in genomic DNA PCR. The pCAMBIA1303 (1303) plasmid was used as a positive control for the PCR reactions with primers of the GUS gene or the kanamycin resistance gene (Kan). (B) Immunoblot analysis of GUS protein and Coomassie blue staining of proteins extracted from root tissues of mock control and A. rhizogenes A8196- or NCPPB 1855-infected plants shown in the upper and lower panels, respectively. The antibody against ß-glucuronidase (GUS protein) was used for immunoblot analysis. (C) Immunoblot analysis of GUS protein and Coomassie blue staining of proteins extracted from roots or leaves of mock control and infected plants. The position of GUS is indicated. NaCl solution used to wash and resuspend bacteria was the mock control. [file 40529_2018_249_MOESM5_ESM.doc]

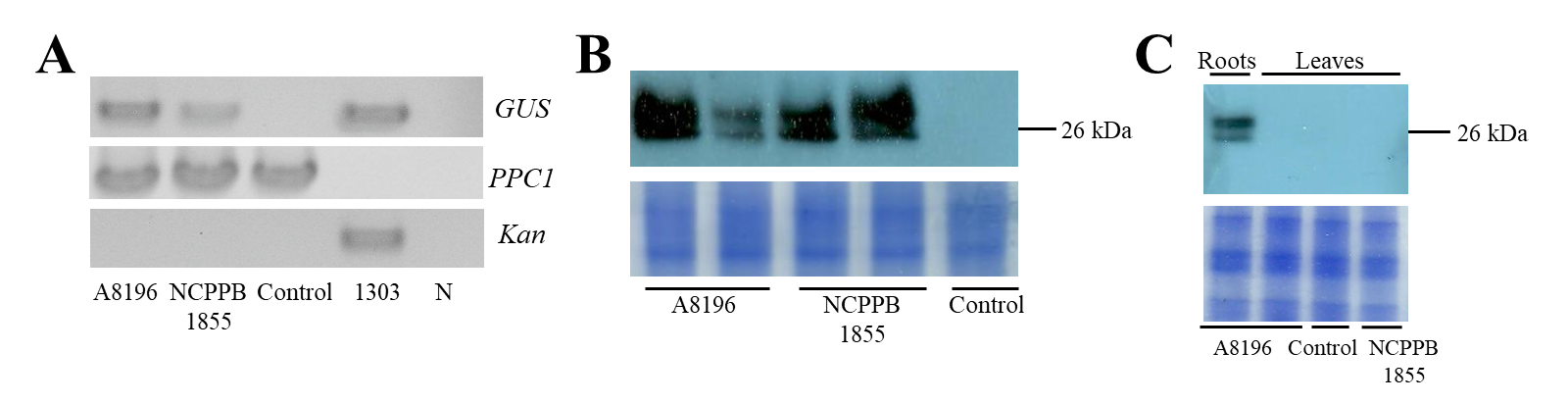


**Figure S2. Genomic DNA PCR results and immunoblot results of hydroponically grown ice plants after infection with the *A. rhizogenes* A8196 or NCPPB 1855 strains harboring the pCAMBIA1303 binary vector.** (A) Genomic DNA was isolated from roots of *A. rhizogenes* A8196- or NCPPB 1855-infected plants infected and uninfected (mock control) plants. Distilled water was a negative control (N) in genomic DNA PCR. The pCAMBIA1303 (1303) plasmid was used as a positive control for the PCR reactions with primers of the GUS gene or the kanamycin resistance gene (*Kan*). (B) Immunoblot analysis of GUS protein and Coomassie blue staining of proteins extracted from root tissues of mock control and *A. rhizogenes* A8196- or NCPPB 1855-infected plants shown in the upper and lower panels, respectively. The antibody against ß-glucuronidase (GUS protein) was used for immunoblot analysis. (C) Immunoblot analysis of GUS protein and Coomassie blue staining of proteins extracted from roots or leaves of mock control and infected plants. The position of GUS is indicated. NaCl solution used to wash and resuspend bacteria was the mock control.
