# Supplementary material for: Effective Agrobacterium-mediated transformation protocols for callus and roots of halophyte ice plant (Mesembryanthemum crystallinum)
Source: Bot Stud. 2019 Jan 7;60:1. doi: 10.1186/s40529-018-0249-3 (PMC6323063; doi:10.1186/s40529-018-0249-3)
Supplement: Supplementary file 6 — Additional file 6: Figure S3. Representative GUS staining results of leaves from hydroponically grown ice plants after infected with the A. rhizogenes A8196 or NCPPB 1855 strains. Leaves of ice plants after infiltration with mock control (Panel A, D), infection with A. rhizogenes A8196 (Panel B, E) or NCPPB 1855 (Panel C, F). Mock control or bacteria infected leaves from plants 8 weeks after treatments are shown in panel A to C. Leaves after GUS staining are shown in panel D to F. Bar = 1 cm. [file 40529_2018_249_MOESM6_ESM.doc]

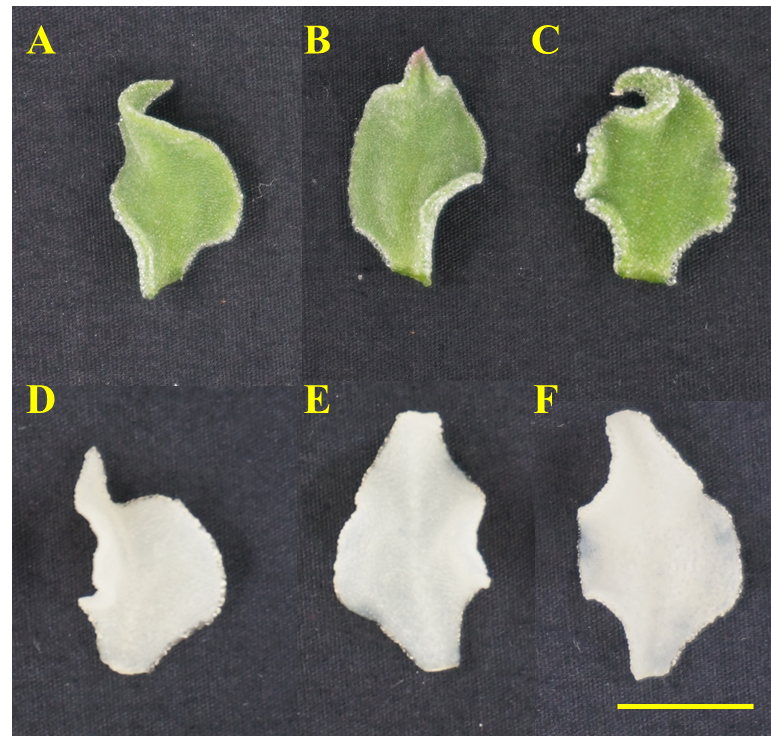


**Figure S3. Representative GUS staining results of leaves from hydroponically grown ice plants after infected with the *A. rhizogenes* A8196 or NCPPB 1855 strains.** Leaves of ice plants after infiltration with mock control (Panel A, D), infection with *A. rhizogenes* A8196 (Panel B, E) or NCPPB 1855 (Panel C, F). Mock control or bacteria infected leaves from plants 8 weeks after treatments are shown in panel A to C. Leaves after GUS staining are shown in panel D to F. Bar = 1cm.
